# Can Ambulatory Blood Pressure Variability Contribute to Individual Cardiovascular Risk Stratification?

**DOI:** 10.1155/2016/7816830

**Published:** 2016-05-09

**Authors:** Annamária Magdás, László Szilágyi, Alexandru Incze

**Affiliations:** ^1^Department for Internal Medicine IV, University of Medicine and Pharmacy of Tîrgu Mureş, Strada Gheorghe Marinescu No. 1, 3rd Floor, 540103 Tîrgu Mureş, Romania; ^2^Faculty of Technical and Human Sciences, Sapientia University of Transylvania, Şoseaua Sighişoarei 1/C, 540485 Tîrgu Mureş, Romania

## Abstract

*Objective*. The aim of this study is to define the normal range for average real variability (ARV) and to establish whether it can be considered as an additional cardiovascular risk factor.* Methods*. In this observational study, 110 treated hypertensive patients were included and admitted for antihypertensive treatment adjustment. Circadian blood pressure was recorded with validated devices. Blood pressure variability (BPV) was assessed according to the ARV definition. Based on their variability, patients were classified into low, medium, and high variability groups using the fuzzy *c*-means algorithm. To assess cardiovascular risk, blood samples were collected. Characteristics of the groups were compared by ANOVA tests.* Results*. Low variability was defined as ARV below 9.8 mmHg (32 patients), medium as 9.8–12.8 mmHg (48 patients), and high variability above 12.8 mmHg (30 patients). Mean systolic blood pressure was 131.2 ± 16.7, 135.0 ± 12.1, and 141.5 ± 11.4 mmHg in the low, medium, and high variability groups, respectively (*p* = 0.0113). Glomerular filtration rate was 78.6 ± 29.3, 74.8 ± 26.4, and 62.7 ± 23.2 mL/min/1.73 m^2^ in the low, medium, and high variability groups, respectively (*p* = 0.0261).* Conclusion*. Increased values of average real variability represent an additional cardiovascular risk factor. Therefore, reducing BP variability might be as important as achieving optimal BP levels, but there is need for further studies to define a widely acceptable threshold value.

## 1. Introduction

Until now, the goal of antihypertensive treatment was to obtain the optimal blood pressure (BP) value defined by international guidelines [1]. However, the blood pressure signal is not stationary; it is characterized by continuous changes during 24-hour intervals, week by week, as well as over longer periods of time like visit-to-visit or within seasons. Although, under physiological conditions, these fluctuations reflect an adaptive response to the everyday stimulus, they may also reflect a disruption in the regulating mechanisms of the cardiovascular (CV) system, which may provide prognostic significance in patients with CV disease [2]. Besides the ability of the 24-hour BP monitoring to evaluate separately daytime and nighttime BP profile, when BP values are known to carry the strongest prognostic value, this approach simultaneously enables us to assess blood pressure variability (BPV) [3, 4]. Studies using ABPM monitoring showed that elevated BPV over 24 hours is associated with the prevalence and progression of target organ damage like impaired renal function, increased left ventricular mass, or left ventricular systolic dysfunction [5–7]. In order to avoid the day-night BP changes, several parameters have been proposed to estimate BPV within 24 hours, but the question has been raised as follows: which one of them is reliable and what is the cut-off point or normal value for that index [8–10]? Therefore, the aim of this study was to assess 24-hour BP profile including its variability and to compare clinical and demographic characteristics of treated hypertensive patients with different degrees of BPV and to establish a normal range for blood pressure variability defined as average real variability (ARV).

## 2. Materials and Methods

In this observational study, a number of 110 treated hypertensive patients were included who were referred to the County Clinical Hospital Tîrgu Mureş to adjust antihypertensive therapy. At the inclusion, from each patient, written informed consent was obtained and approved by the Local Ethic Committee, according to the International Ethical Guidelines and Declaration of Helsinki. Inclusion criteria were diagnosis of hypertension based on ABPM with mean BP values greater than 130/80 mmHg and ability to sign informed consent. Patients with cardiac arrhythmias, congestive heart failure NYHA class III/IV, and type 1 diabetes mellitus, shift workers, and pregnant women were excluded from the study.

Circadian BP profile was assessed with validated devices (ABPM 05® and cardXplore, Meditech Ltd., Hungary) applied on the nondominant arm of the patient. Monitoring began between 8 and 10 am and measurements frequency was set at 20 minutes daytime (06:00–21:59) as well as nighttime (22:00–05:59). Daytime and nighttime systolic/diastolic BP values, pulse pressure, diurnal/nocturnal index, morning surge, and standard deviation were provided automatically by the measurement device. The ABPM validation criteria were the presence of at least 70% of the scheduled measurements and at least 48 BP values over 24 hours [11]. The 24-hour BP variability was calculated using the formula of average real variability:

(1) $$\begin{array}{c} ARV=\frac{1}{N-1}\overset{N-1}{\underset{k=1}{\sum }}\left|\;{BP}_{k+1}-{BP}_{k}\;\right|, \end{array}$$

where *N* represents the number of BP measurements in a given subject and BP_*k*_ is the blood pressure at measurement number *k* [8]. The alternative parameter that characterizes blood pressure variability is the standard deviation of the 24-hour systolic BP, calculated according to the formula as follows:

(2) $$\begin{array}{c} SD=\sqrt{\frac{1}{N-1}\overset{N}{\underset{k=1}{\sum }}\left|{BP}_{k+1}-\bar{BP}\right|}, \end{array}$$

where *N* is the number of valid BP measurements and $\bar{BP}$ is the average of ABPM readings [12]. The estimated glomerular filtration rate was assessed by the formula of MDRD (Modification of Diet in Renal Disease Study) [13]:

(3) eGFR=141×0.993Agemin⁡Scr/0.9,10.411×max⁡Scr/0.9,11.209if  male143.5×0.993Agemin⁡Scr/0.7,10.329×max⁡Scr/0.7,11.209if  female,

where *S*_cr_ is serum creatinine in mg/dL. One factor concerning the race of the patient was omitted from the above formula, as it did not hold for any of the patients.

After calculating ARV, the fuzzy *c*-means (FCM) algorithm [14] was applied to divide patients into low, medium, and high variability groups. Employing a clustering algorithm instead of dividing patients according to percentiles was preferred, because theoretically this approach is able to establish optimal boundaries, which assure that individuals placed in the same class are most similar and separated ones are most dissimilar.

In order to assess cardiovascular risk, laboratory analysis was performed. Data were collected as raw data and statistical analysis was performed using Matlab. Numerical data are represented as mean ± SD. Means were compared using one-way ANOVA test and Chi-square test for categorical variables. To assess the individual contribution of various risk factors, multivariate linear regression was employed. A *p* value less than 0.05 was considered statistically significant with a confidence interval of 95%.

## 3. Results

**Figure 1:**
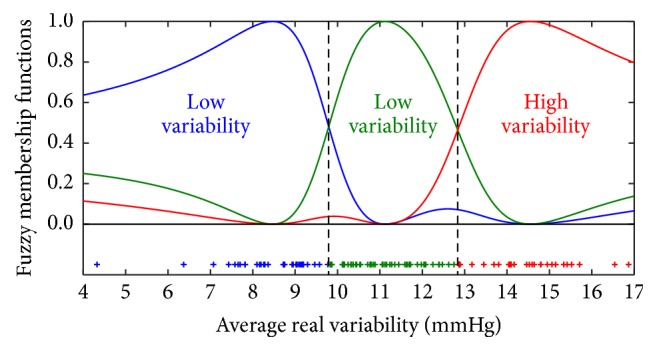
Fuzzy membership functions produced by the fuzzy *c*-means algorithm for the three ARV classes. Individual ARV values are plotted along the horizontal axis.

Based on the 24-hour systolic BP variability defined with ARV, fuzzy membership functions and patient groups were obtained by the FCM algorithm as presented in Figure 1. Patients were classified into three groups labeled as low, medium, or high variability (LV, MV, or HV): the threshold between LV and MV was established at 9.8 mmHg, while the boundary between MV and HV was established at 12.8 mmHg.

**Figure 2:**
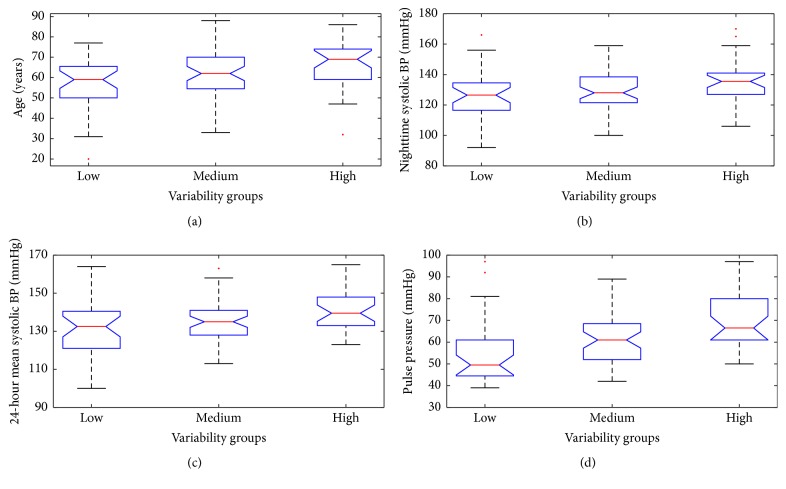
ANOVA test for age, nighttime, and 24-hour mean systolic BP and pulse pressure.

The characteristics of the groups are summarized in Table 1. Four of the statistically significant differences are plotted in Figure 2 as follows. The mean age of the patients showed statistically significant difference among the groups, with 56.4 ± 13.6 versus 62.0 ± 11.1 versus 67.1 ± 11.4 years in the low, medium, and high variability groups, respectively (*p* = 0.0028). The highest values of the systolic blood pressure were found in the HV group. The highest pulse pressure was also recorded in the HV group with 69.7 ± 13.2 mmHg, compared to 60.5 ± 11.4 mmHg in the MV, and 55.8 ± 15.5 mmHg in the LV group (*p* = 0.0003). Further significant differences were recorded in daytime systolic BP, systolic BP standard deviation, and the estimated glomerular filtration rate (Table 1).

Multivariate linear regression was employed to assess the contribution of various possible risk indicators to the characterization of cardiovascular risks. Standardized regression coefficients and corresponding *p* values are reported in Tables 2 and 3.

Tables 2 and 3 allow us to assess and compare the usefulness of blood pressure variability related parameters ARV and SD. SD can be high in case of patients who have relatively high BP during daytime and relatively low BP during nighttime, because all measured values are compared to the mean BP. On the other hand, ARV, by reflecting the differences between consecutive measurements, can assess rapid fluctuations of the blood pressure. These tables clearly show the significance of ARV in the variation of mean systolic BP and pulse pressure, which is not the case for SD, demonstrating that ARV indeed can contribute to the stratification of cardiovascular risk factors.

Tables 2 and 3 also reveal that the age of the patient is the main risk indicator in case of chronic kidney disease; high pulse pressure and low diastolic BP are also mostly age-related, which is in fact caused by the increased stiffness of artery walls. Further on, according to these tables, higher BMI can be associated with the risk of diabetes and high diastolic BP.

## 4. Discussion

Although blood pressure variability defined with ARV and SD showed statistically significant difference between the groups, ARV could represent a more reliable parameter for defining variability groups, because it takes into account the order in which measurements were performed, reflecting the absolute value of the differences between consecutive measurements, while SD reflects the BP data spread around a central value and does not consider the order in which the BP measurements were collected. In accordance with other researchers, we found that patients with different BP data sets could have identic SD but are placed in different variability groups based on their ARV [8]. Subjects in the high variability group were older, 8 of them had diabetes mellitus, and they presented the lowest glomerular filtration rate. Regarding the ABPM data, daytime, nighttime, and mean systolic blood pressure as well as pulse pressure—as a marker of arterial stiffness—were greater in this group. After classification of the patients based on their variability, we also defined three groups based on their age. Thereby, maximum limit for the young group was 55 years, middle age was between 56 and 69 years, and patients older than 70 years were classified as elderly. Group sizes were almost the same as in case of ARV clusters. According to age based classification, in the elderly group only the estimated glomerular filtration rate and pulse pressure were significantly higher compared to the other age categories. These results let us conclude that an increase in blood pressure variability is not just the consequence of age; however, it could be also related to high sodium intake or altered baroreflex function [15].

In a study performed on over 8000 hypertensive subjects, ARV over 24 hours was a better predictor of mortality as well as cardiovascular events and stroke, compared to 24-hour SD [12]. Other studies recommend the use of variability parameters based on 24-hour monitoring that does not include BP levels [16, 17]. Although there are different points of views regarding the contribution of BP variability to cardiovascular risk stratification as well as the selection of the variability index, noninvasive assessment of 24-hour BP should be performed in all hypertensive patients and calculation of variability using ARV should be preferred over SD.

Thus, hypertensive subjects with high BP variability (ARV) display greater cardiovascular risk compared to those with normal variability. In contrast to the study conducted by Mena et al., where high variability was defined as ARV exceeding 9.86 mmHg, in our study population the threshold value was 12.8 mmHg [8]. In our study we investigated a small group of hypertensive subjects under antihypertensive medication; a reason why our results differ from those observed by Mena et al. could be that we also included patients with diabetes mellitus type 2 and patients with chronic kidney disease. This could be a possible explanation of why the threshold value for high variability group is increased. Given the fact that the cut-off value of 9.8 mmHg is common to both studies, it could represent a starting point for further studies meant to define a widely acceptable threshold value for high variability.

## 5. Conclusion

The assessment of ambulatory monitoring derived blood pressure variability could represent an additional cardiovascular risk factor in hypertensive patients and could be of importance in individual risk stratification. Therefore, in high risk hypertensive patients, lowering of BP variability might be as important as achieving optimal BP levels. Although there are different threshold values to define high variability, there is need for a widely accepted value.

## Figures and Tables

**Table 1:** Characteristics of the three studied ARV groups.

Characteristics	Unit	Low variability ARV < 9.8 mmHg	Medium variability ARV between 9.8 and 12.8 mmHg	High variability ARV > 12.8 mmHg	*p* value
Male/female	(Number)	12/20	22/26	8/22	0.32
Age	(Years)	56.4 ± 13.6	62.0 ± 11.1	67.1 ± 11.4	**0**.**0028**
BMI	(kg/m^2^)	28.9 ± 4.3	30.5 ± 5.9	30.7 ± 5.3	0.31
DM type 2	(Number)	3	6	8	>0.05
CKD	(Number)	5	9	2	>0.05
Blood sugar	(mg/dL)	105.1 ± 25.8	112.5 ± 48.2	111.8 ± 34.1	0.68
Triglycerides	(mg/dL)	164.4 ± 68.5	191.8 ± 112.4	151.4 ± 54.1	0.12
Total cholesterol	(mg/dL)	197.3 ± 63.1	204.6 ± 63.3	196.1 ± 43.7	0.78
eGFR	(mL/min/1.73 m^2^)	78.6 ± 29.3	74.8 ± 26.4	62.7 ± 23.2	**0**.**0261**
ACEI/ARBs	(Number)	18/2	30/11	21/4	0.19
CCB	(Number)	14	28	19	0.67
BB	(Number)	14	28	15	0.32
Diuretics	(Number)	28	29	31	>0.05
Daytime sBP	(mmHg)	133.7 ± 16.6	137.6 ± 14.1	144.6 ± 11.2	**0**.**011**
Daytime dBP	(mmHg)	78.0 ± 12.1	76.8 ± 11.0	75.1 ± 10.2	0.59
Nighttime sBP	(mmHg)	125.6 ± 17.9	130.0 ± 13.5	135.3 ± 15.4	**0**.**0478**
Nighttime dBP	(mmHg)	69.6 ± 10.4	69.4 ± 9.5	66.3 ± 10.2	0.33
Mean sBP	(mmHg)	131.2 ± 16.7	135.0 ± 12.1	141.5 ± 11.4	**0**.**0113**
Mean dBP	(mmHg)	75.4 ± 11.0	74.5 ± 9.6	71.8 ± 9.3	0.35
Morning surge	(mmHg)	19.4 ± 10.7	20.5 ± 10.8	20.2 ± 12.3	0.91
D/ND	(Number)	13/19	16/32	12/18	0.22
Heart rate	(Beat/min)	67.2 ± 10.6	65.7 ± 12.2	64.6 ± 9.3	0.66
PP	(mmHg)	55.8 ± 15.5	60.5 ± 11.4	69.7 ± 13.2	**0**.**0003**
sBP SD	(mmHg)	12.5 ± 2.4	13.7 ± 2.7	15.0 ± 3.3	**0**.**0027**

BMI: body mass index, DM: diabetes mellitus, CKD: chronic kidney disease, eGFR: estimated glomerular filtration rate, ACEI: angiotensin converting enzyme inhibitor, ARBs: angiotensin receptor blockers, CCB: calcium channel blockers, BB: beta blockers, sBP/dBP: systolic/diastolic blood pressure, D: dipper, ND: nondipper, PP: pulse pressure, and SD: standard deviation.

**Table 2:** Standardized regression coefficients and *p* values given by multivariate regression, using input variables age, ARV, BMI, and gender.

Outcome	Age		ARV		BMI		Gender	
Variable	Coefficient	*p* value	Coefficient	*p* value	Coefficient	*p* value	Coefficient	*p* value
eGFR	−**0**.**236**	**0**.**019**	0.020	0.84	0.118	0.22	0.154	0.11
Daytime sBP	0.014	0.88	**0**.**292**	<**0**.**005**	0.141	0.13	0.098	0.30
Nighttime sBP	0.129	0.21	0.145	0.16	0.051	0.60	0.030	0.76
Mean SBP	0.094	0.36	**0**.**235**	**0**.**02**	0.105	0.27	0.081	0.40
Pulse pressure	**0**.**404**	<**0**.**001**	**0**.**193**	**0**.**036**	−0.018	0.84	0.005	0.96
Daytime dBP	−**0**.**462**	<**0**.**001**	0.110	0.23	0.145	0.10	0.095	0.28
Nighttime dBP	−**0**.**374**	<**0**.**001**	−0.016	0.87	0.155	0.09	0.095	0.30
Mean dBP	−**0**.**446**	<**0**.**001**	0.054	0.55	**0**.**171**	**0**.**05**	0.106	0.23
DM type 2	−0.011	0.92	0.149	0.14	0.179	0.066	−0.103	0.28

**Table 3:** Standardized regression coefficients and *p* values given by multivariate regression, using input variables age, SD, BMI, and gender.

Outcome	Age		SD		BMI		Gender	
Variable	Coefficient	*p* value	Coefficient	*p* value	Coefficient	*p* value	Coefficient	*p* value
eGFR	−**0**.**239**	**0**.**014**	0.067	0.50	0.102	0.30	0.150	0.12
Daytime sBP	0.068	0.47	**0**.**253**	**0**.**011**	0.120	0.22	0.084	0.38
Nighttime sBP	**0**.**193**	**0**.**05**	−0.176	0.08	0.132	0.19	0.044	0.65
Mean SBP	0.150	0.13	0.076	0.45	0.126	0.21	0.079	0.42
Pulse pressure	**0**.**454**	<**0**.**001**	0.055	0.55	0.021	0.98	0.003	0.97
Daytime dBP	−**0**.**451**	<**0**.**001**	0.166	0.065	0.116	0.20	0.085	0.33
Nighttime dBP	−**0**.**354**	<**0**.**001**	−**0**.**205**	**0**.**026**	**0**.**214**	**0**.**02**	0.109	0.22
Mean dBP	−**0**.**434**	<**0**.**001**	0.028	0.75	**0**.**173**	**0**.**05**	0.105	0.24
DM type 2	0.027	0.79	0.051	0.61	0.191	0.006	−0.105	0.28

## References

[1] Mancia G., Fagard R., Narkiewicz K. (2013). 2013 ESH/ESC Guidelines for the management of arterial hypertension: the Task Force for the management of arterial hypertension of the European Society of Hypertension (ESH) and of the European Society of Cardiology (ESC). *Journal of Hypertension*.

[2] Parati G., Ochoa J. E., Lombardi C., Bilo G. (2015). Blood pressure variability: assessment, predictive value, and potential as a therapeutic target. *Current Hypertension Reports*.

[3] Hermida R. C., Smolensky M. H., Ayala D. E. (2013). 2013 Ambulatory blood pressure monitoring recommendations for the diagnosis of adult hypertension, assessment of cardiovascular and other hypertension-associated risk, and attainment of therapeutic goals. *Chronobiology International*.

[4] Parati G., Ochoa J. E., Salvi P., Lombardi C., Bilo G. (2013). Prognostic value of blood pressure variability and average blood pressure levels in patients with hypertension and diabetes. *Diabetes Care*.

[5] Tatasciore A., Renda G., Zimarino M. (2007). Awake systolic blood pressure variability correlates with target-organ damage in hypertensive subjects. *Hypertension*.

[6] Manios E., Tsagalis G., Tsivgoulis G. (2009). Time rate of blood pressure variation is associated with impaired renal function in hypertensive patients. *Journal of Hypertension*.

[7] Tatasciore A., Zimarino M., Tommasi R. (2013). Increased short-term blood pressure variability is associated with early left ventricular systolic dysfunction in newly diagnosed untreated hypertensive patients. *Journal of Hypertension*.

[8] Mena L., Pintos S., Queipo N. V., Aizpúrua J. A., Maestre G., Sulbarán T. (2005). A reliable index for the prognostic significance of blood pressure variability. *Journal of Hypertension*.

[9] Mancia G., Bombelli M., Facchetti R. (2007). Long-term prognostic value of blood pressure variability in the general population: results of the Pressioni Arteriose Monitorate e Loro Associazioni study. *Hypertension*.

[10] Bilo G., Giglio A., Styczkiewicz K. (2007). A new method for assessing 24-h blood pressure variability after excluding the contribution of nocturnal blood pressure fall. *Journal of Hypertension*.

[11] Mena L. J., Maestre G. E., Hansen T. W. (2014). How many measurements are needed to estimate blood pressure variability without loss of prognostic information?. *American Journal of Hypertension*.

[12] Hansen T. W., Thijs L., Li Y. (2010). Prognostic value of reading-to-reading blood pressure variability over 24 hours in 8938 subjects from 11 populations. *Hypertension*.

[13] Levey A. S., Stevens L. A., Schmid C. H. (2009). A new equation to estimate glomerular filtration rate. *Annals of Internal Medicine*.

[14] Bezdek J. C. (1981). *Pattern Recognition with Fuzzy Objective Function Algorithms*.

[15] Simmonds S. S., Lay J., Stocker S. D. (2014). Dietary salt intake exaggerates sympathetic reflexes and increases blood pressure variability in normotensive rats. *Hypertension*.

[16] Yamaguchi Y., Wada M., Sato H. (2014). Impact of ambulatory blood pressure variability on cerebral small vessel disease progression and cognitive decline in community-based elderly Japanese. *American Journal of Hypertension*.

[17] Rothwell P. M., Howard S. C., Dolan E. (2010). Effects of *β* blockers and calcium-channel blockers on within-individual variability in blood pressure and risk of stroke. *The Lancet Neurology*.

